# Effects of yeast culture on growth performance, antioxidant capacity, immune function, and intestinal microbiota structure in Simmental beef cattle

**DOI:** 10.3389/fvets.2024.1533081

**Published:** 2025-01-31

**Authors:** Xueqiang Li, Nan An, Hui Chen, Dacheng Liu

**Affiliations:** ^1^College of Veterinary Medicine, Inner Mongolia Agricultural University, Hohhot, China; ^2^Key Laboratory of Clinical Diagnosis and Treatment of Animal Diseases, Ministry of Agriculture, Hohhot, China

**Keywords:** yeast culture, growth performance, antioxidant capacity, immune function, intestinal microbiota structure

## Abstract

**Introduction:**

As functional feed additives, yeast cultures have been applied in animal husbandry and shown a wide range of good efficacy. This paper aimed to evaluate the effects of yeast culture (YC) on the growth performance, antioxidant capacity, immune function, and intestinal microbiota structure in beef cattle.

**Methods:**

Forty Simmental bulls were randomly divided into two groups, including the control group fed with Total mixed ration (TMR) and YC group fed with TMR supplemented with 2% YC, for 60 days. Serum samples were collected on the 1st, 30th, and 60th days, respectively, while feces 3 days before the end of the test.

**Results:**

Results showed that YC addition significantly elevated average daily gain and reduced feed to weight ratio of beef cattle. The enzyme activities of total superoxide dismutase, total antioxidant capacity, and glutathione peroxidase in the serum in YC group obviously increased, while the malondialdehyde content distinctly decreased. Furthermore, YC feeding significantly enhanced the immunoglobulin G (IgG), IgA, IgM levels, lysozyme content, alkaline phosphatase activity, as well as the contents of interleukin-1β (IL-1β), IL-6, and interferon-γ in the cattle serum. The Shannon and Observed species indexes of fecal samples in YC group were remarkably higher than those in the control group, with the former group exhibiting a significant increase in the relative abundance of *Paraprevotellace_CF231* and *Peptostreptococcaceae_Clostridium* at the genus level, while the relative abundance of *Spirochaetaceae_Treponema* decreased significantly. Moreover, the abundance of *Clostridium* and *CF231* was positively associated with the levels of serum antioxidant capacity and immune function indicator contents.

**Discussion:**

In conclusion, YC could obviously improve the growth performance, antioxidant capacity, immune function, and intestinal microbiota structure in Simmental beef cattle. These results provide a theoretical basis for the clinical application of such yeast fermented preparations in beef cattle husbandry.

## 1 Introduction

The growth period is critical in beef cattle breeding, during which the animal health state and growth performance directly affect the length of the breeding cycle and the production efficiency as well. Therefore, during this period, it is a hot topic to research how to improve their resistance to diseases and growth performance. In clinical practice, various forage additives such as antibiotics have been commonly used to promote production performance by increased food intake and weight gain and improved herd health (1). However, antibiotic application in animal feeds is frequently questioned since they have been proved to result in serious complications due to drug resistance and their residues in the animal products (2). Consequently, finding suitable alternative strategies to antibiotics is required, and the common ones are probiotics and their cultures, among which yeast cultures (YCs) are showing more and more promising effects (3).

Multiple studies have shown that YCs have a great deal of biological effects in various animal species, as they can promote the growth performance, immune function and antioxidative capacity, and can regulate inflammatory factors (4–6). For ruminants, YCs could regulate rumen fermentation, promote nutrient digestion and absorption, and maintain microecological balance of the body (4, 7, 8). Furthermore, YCs can ameliorate the physiological structure of the intestine, stimulate its development, and modify its microbiota structure as well (9).

At present, many probiotic fermented products for ruminants have emerged, but their quality and usage effects vary largely. YC is a microecological preparation previously developed by our research group specifically for ruminants, which contains a small number of live yeast cells and a quantity of metabolites. The two dominant yeast strains have shown significant advantages in cell biomass, enzyme production ability, and especially in the ability to secrete active substances. Using these two yeasts as fermentation strains, YC was produced in a specific culture medium through special fermentation processes (10). We have conducted research on mutton sheep such as Mongolian sheep, and the results showed that YC can significantly improve the growth performance and economic benefits and promote the immune function and antioxidant capacity (10). However, due to various factors such as individual characteristics of species and difficulty in sampling implementation, there are few reports on YC study in beef cattle. Therefore, in order to explore whether YC could be a potential alternative to growth-promoting and disease-preventing antibiotics, we used hybrid Simmental beef cattle to study the effects of YC on their growth performance, antioxidant capacity, immune function, and intestinal microbiota structure.

## 2 Materials and methods

### 2.1 YC preparation

According to the preliminary research results of our team (11, 12), strains of *Saccharomyces cerevisiae* and *Kluyveromyces marxianus* exhibiting good fermentation characteristics were isolated from naturally fermented horse milk. Taken these two yeasts as fermentation strains, 28% corn germ meal, 12% spraying corn bran, 12% bran, 10% rice bran, 10% soybean meal, 10% corn, 10% cottonseed meal, and 8% red dog wheat raw materials were mixed to prepare solid-state fermentation medium. One:one mixture of the two yeasts (3 × 10^8^ cfu/g) were inoculated at a concentration of 8% per 1,000 kg wet mixed matrix, with the addition of sterile water while stirring, resulting in a total moisture content in the system of 40%. Aerobic fermentation was then conducted for 72 h at 30°C. The main nutritional contents of YC are as follows: crude protein ≥ 18.0%, moisture ≤ 12.0%, crude ash ≤ 9.0%, mannan ≥ 0.5%, number of active yeast ≥ 10^6^ cfu/g (13).

### 2.2 Animals and diet

Animal experiment was conducted in accordance with the Animal Experiment Guidelines of the National Institute of Animal Health (GB 14925-2010) and approved by the Ethics Committee of Inner Mongolia Agricultural University (NND2022072). This experiment was conducted on a beef cattle farm in Horqin District, Tongliao City, Inner Mongolia Autonomous Region, China, with geographical coordinates between 43° 22′-43° 58′ N and 121° 42′-123° 02′ E, from September to December 2022. Forty healthy Simmental bulls aged 150 ± 7 days with a body weight of 200 ± 8.06 kg were randomly divided into two groups, with 20 replicates in each group. The cattle in the control group were fed with a Total mixed ration (TMR), while those in the test group were fed with TMR supplemented with 2% of YC. The test period was totally 67 days, of which the pre-trial period was 7 days. During the pre-trial period, beef cattle were inoculated, dewormed and ear-labeled, and the cattle sheds were disinfected, according to the routine procedures of the cattle farm. The cattle were fed three times per day, at 04:20, 11:00, and 15:00, respectively, free access to water.

The TMR diet of beef cattle was prepared according to the National Academies of Science, Engineering, and Medicine (47) standard, and the compositions and nutritional levels of the feed are shown in Table 1. The determination methods for nutritional levels of dry matter (DM), crude protein (CP), neutral detergent fiber (NDF), and acidic detergent fiber (ADF) refer to GB/T 6435-2014, GB/T 6432-2018, GB/T 20806-2006, and NY/T 1459-2007, respectively.

**Table 1:** Ingredient composition and nutrient levels of diets (DM basis).

**Items**	**Control group**	**YC group**
**Ingredients, %**		
Whole corn silage	19.98	19.98
Corn stalk	18.54	18.04
*Leymus chinensis*	12.88	12.38
Corn meal	6.42	5.82
Soybean meal	5.65	5.65
Flaked corn	3.02	3.02
Wheat bran	1.51	1.31
NaHCO_3_	0.38	0.38
NaCl	0.38	0.38
Granular concentrated feed	29.73	29.53
5% Premix^a^	1.51	1.51
Yeast culture		2.00
Total	100.00	100.00
**Nutrient levels** ^b^		
DE/(MJ/kg)	9.91	9.93
ME/(MJ/kg)	9.49	9.52
DM	77.16	77.13
CP	11.35	11.33
ADF	25.18	25.25
NDF	37.89	37.93
Ca	0.611	0.621
P	0.386	0.383

^a^ The premix provided the following per kg of diets: VA 150,000 IU, VD3 30,000 IU, VE 600 IU, VB1 10 mg, VB2 28 mg, VB6 10 mg, VB12 100 μg, Biotin 2 mg, Folic acid 4 mg, D-pantothenic acid 50 mg, Nicotinic acid 200 mg, Cu 0.2 g, Fe 3 g, Mn 2 g, Zn 1.2 g, I 3 g, Se 1 g.

^b^ DE and ME are calculated values, while the others are measured values.

### 2.3 Sample collection

#### 2.3.1 Collection of serum samples

Ten cattle were selected from the control group and six from the test group, and 10 ml blood was collected from the jugular vein using aseptic needles and vacuum biochemical tubes, before morning feeding, on the 1st, 30th, and 60th days of the trial period. After the blood samples were kept resting for 30 min and centrifuged at 4,000 r/min for 10 min, the upper serum was sucked out using a disposable straw and stored at −20°C in a sterile, enzyme-free cryopreserved tube for subsequent measurement of immune function and antioxidant capacity indexes.

#### 2.3.2 Collection of fecal samples

Three days before the end of the test, eight uncontaminated fresh fecal samples randomly collected from eight cattle in each group were placed in a 5 mL sterile frozen tube, stored in liquid nitrogen and then at −80°C for future measurement. The samples were sent to Shanghai Pinozen Biotechnology Co., Ltd. through dry ice to conduct the detection of intestinal microbiota structure.

### 2.4 Index measurement and methods

#### 2.4.1 Measurement of growth performance indexes

The daily feeding amounts were recorded and the remaining parts were weighed before feeding of the next day to calculate the average daily feed intake (ADF). During the formal trial period, the fasting weights were measured before morning feeding on the 1st, 30th, and 60th days, and the average daily gain (ADG) and feed to weight ratio (F/G) were calculated.

The relevant calculation formulas are as follows:

ADF (kg/d) = [total feed amounts (kg)–total residue amounts (kg)]/trial days
ADG (kg/d) = (final body weights–initial body weights)/trial days
Feed to Weight Ratio = Dry Matter Intake (DMI)/ADG
DMI (kg) = ADF ^*^ dry matter content of basic diet

#### 2.4.2 Determination of antioxidant capacity indexes

The total superoxide dismutase (T-SOD) activity in cattle serum was measured using the hydroxylamine method. The activity of glutathione peroxidase (GSH-Px) was estimated with the dithiodinitrobenzoic acid (DTNB) colorimetric method. The total antioxidant capacity (T-AOC) level was measured using colorimetric method. The malondialdehyde (MDA) content was determined by the thiobarbituric acid method. The determination kits for above indexes were all purchased from Nanjing Jiancheng Biotechnology Research Institute, referring to the instructions of the reagent kit for the specific experimental procedure. The information of the kits is presented in Table 2.

**Table 2:** Serum antioxidant index kits and their product numbers.

**Items**	**Kit names**	**Product numbers**
T-SOD	Total superoxide dismutase (T-SOD) assay kit (Hydroxylamine method)	A001-1-1
T-AOC	Total antioxidant capacity (T-AOC) assay kit	A015-1-1
MDA	Malondialdehyde (MDA) assay kit (TBA method)	A003-1-1
GSH-Px	Glutathione Peroxidase (GSH-Px) assay kit	A005-1-1

#### 2.4.3 Determination of immune function indexes

The serum content of lysozyme (LZM) was evaluated by the turbidimetric inhibition immunoassay. The alkaline phosphatase (AKP) activity was determined using micro-enzyme labeling method. The serum levels of immunoglobulin and cytokines, including immunoglobulin G (IgG), immunoglobulin A (IgA), immunoglobulin M (IgM), interleukin-1β (IL-1β), IL-6, and interferon-γ (IFN-γ), were assessed with enzyme-linked immunosorbent assay (ELISA) kits. The above testing kits were all purchased from Nanjing Jiancheng Biotechnology Research Institute, referring to the instructions of the reagent kit for the specific experimental procedure. The information of the kits is presented in Table 3.

**Table 3:** Serum immune index kits and their product numbers.

**Items**	**Kit names**	**Product numbers**
IgG	Immunoglobulin G assay kit	H106-1-1
IgA	Immunoglobulin A assay kit	H108-1-2
IgM	Immunoglobulin M assay kit	H109-1-1
LZM	Lysozyme assay kit	A050-1-1
AKP	Alkaline phosphatase (AKP) assay kit	A059-3-1
IL-1β	Interleukin-1β assay kit	H002-1-1
IL-6	Interleukin-6	H007-1-1
IFN-γ	Interferon-γ assay kit	H025-1-1

#### 2.4.4 Bacterial 16s rRNA gene high-throughput sequencing

Genomic DNA from the microbial community in the fecal samples was extracted using the OMEGA Soil DNA Kit (M5635-02, OMEGA Bio-Tek, Norcross, GA, USA) according to the manufacturer's protocol. The DNA concentration was quantified by NanoDrop ND-1000 spectrophotometer (Thermo Fisher Scientific, Waltham, MA, USA), and the purity of the extracted DNA was checked by 1.2% agarose gel electrophoresis. The primer sequences used to amplify the V3–V4 region of bacteria are: F: 5′- CTCTACGGGAGGCAGCAG−3′ and R: 5 '- GGACTACHVGGGTWTCTAAT−3′. Meanwhile, sample-specific 7-bp barcode was combined into the primers for multiple sequencing. PCR amplification was performed with Pfu high-fidelity DNA polymerase from TransGen Biotech. The amplification program was as follow: 96°C for 6 min, 28 cycles at 96°C for 30 s, 56°C for 30 s, and 72°C for 45 s, followed by a hold at 72°C for 10 min and preservation at 4°C. The PCR amplification products were purified by Vazyme VAHTSTM DNA Clean Beads (Vazyme, Shanghai, China), and fluorescence quantification was performed using Microplate reader instrument (BioTek, FLx800) and Quant-iT PicoGreen dsDNA Assay Kit (Invitrogen, Carlsbad, CA, USA). The samples were processed according to the results and sequencing requirements. Paired-end sequencing was performed using NovaSeq sequencer on an Illumina MiSeq platform with NovaSeq6000 SP kit (500 cycles) from Biotechnology Co., Ltd. (Shanghai, China), and the sample sequencing depth was 50,000 Tags. The raw reads were uploaded to the NCBI Sequence Read Archive (SRA) database (entry number: PRJNA1038692).

The data were subjected to Vsearch software for de-primer, splicing, quality-filtering, de-duplication, de-chimerism, and clustering (14), after which the characteristic sequences of each sample were obtained. Based on 97% sequence similarity for clustering, the Greengenes database was used to partition and identify the operational classification units (OTUs) using QIIME2 (2019.4) software (15). Furthermore, QIIME2 (2019.4) software was also used to perform several key analyses, such as α diversity analysis (including Chao1, Observed species, Shannon, and Simpson), β diversity analysis (including PCoA analysis), species composition, etc.

### 2.5 Statistical analysis

With individual bulls as experimental units, the data were analyzed using a completely random design. Meanwhile, the Durbin Watson test was used to check the randomness of initial and final body weight data to verify the effectiveness of randomization. The data were organized and calculated using Excel 2010. Furthermore, the data concerned on the indexes of growth performance, antioxidant capacity and immune function were analyzed by the built-in *T*-test analysis method in the IBM SPSS.22 software system. The results were expressed as “Mean ± standard error,” with *P* < 0.05 indicating significant differences and *P* < 0.01 indicating extremely significant differences.

## 3 Results

### 3.1 Effects of YC on the growth performance of beef cattle

According to Table 4, the ADF levels of control group and YC group presented no significant difference during the period of 1st to 30th days (*P* > 0.05). On the other hand, the ADF in YC group increased by 5.50% by an average of 0.6 kg per cow per day during the 30th to 60th days, compared to that in the control group (*P* < 0.01). However, from the perspective of throughout the entire test period, concerning on the ADF amounts, there was no significant difference between the two groups, but increasing by 3.44% in the test group (*P* > 0.05). Furthermore, the ADG in YC group was obviously upregulated by 9.09% compared to the control group (*P* < 0.05), while the F/G ratio was downregulated by 5.23% (*P* > 0.05), during the 1st to 60th days. Specifically, the ADG level rose by 6.36% and 11.36%, respectively, during the periods of 1st to 30th and 30th to 60th days, while F/G declined by 5.49% and 5.33%, respectively, during the same trial periods (*P* > 0.05).

**Table 4:** Effects of YC on the growth performance of beef cattle.

**Items**	**Time points**	**Control group**	**YC group**	***P*-value**
ADF (kg/d)	Day 1–30	8.29 ± 0.73	8.36 ± 0.78	0.71
	Day 31–60	10.91 ± 0.71	11.51 ± 0.88^**^	0.005
	Day 1–60	9.60 ± 1.50	9.93 ± 1.78	0.27
ADG (kg/d)	Day 1–30	1.10 ± 0.25	1.17 ± 0.21	0.45
	Day 31–60	1.32 ± 0.21	1.47 ± 0.19^*^	0.04
	Day 1–60	1.21 ± 0.15	1.32 ± 0.13^*^	0.02
F/G	Day 1–30	5.83 ± 0.53	5.51 ± 0.53	0.53
	Day 31–60	6.38 ± 0.47	6.04 ± 0.53	0.45

^*^p < 0.05, ^**^p < 0.01. The value is the mean ± SD, n = 20.

ADG, average daily gain.

ADF, average daily feed intake.

F/G, ADF/ADG.

### 3.2 Effects of YC on antioxidant capacity of beef cattle

**Figure 1:**
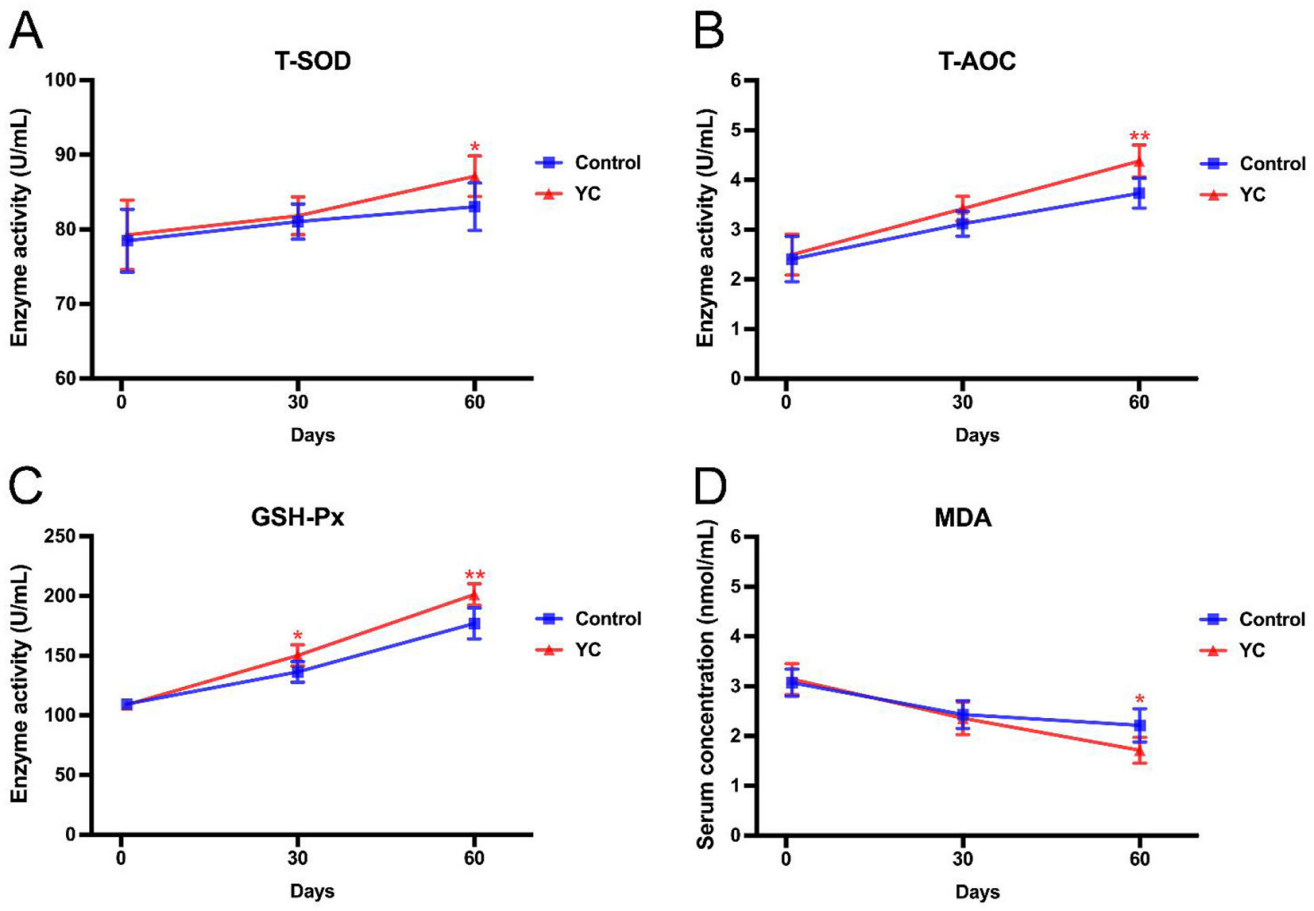
Effects of YC on the indexes related to antioxidant capacity of beef cattle. **(A)** Effects on the serum T-SOD activity. **(B)** Effects on the serum T-AOC activity. **(C)** Effects on the serum GSH-Px activity. **(D)** Effects on the serum MDA concentration. *p < 0.05, **p < 0.01. The value is the mean ± SD, *n* = 6.

From Figure 1, it can be found that at the beginning of the experiment (on the 1st day), all the four indexes of serum antioxidant capacity of beef cattle in the control group and YC group were separately at the similar levels (*P* > 0.05). On the 30th day, the T-SOD activities, T-AOC levels, and MDA contents in the two groups were still with no significant difference, while GSH-Px activity in the YC group was obviously higher than that in the control group. On the 60th day, the T-SOD and GSH-Px activities as well as T-AOC levels in the cattle serum of YC group showed varying degrees of obvious increases, by 4.93%, 13.70%, and 17.43%, respectively (*P* < 0.05). On the contrary, the MDA content in YC group was remarkably reduced by 22.62% at the same time point (*P* < 0.05). The above results indicated that as the YC feeding time prolonged, its promoting efficacy on the antioxidant capacity of beef cattle appeared more and more significant.

### 3.3 Effects of YC on immune function of beef cattle

**Figure 2:**
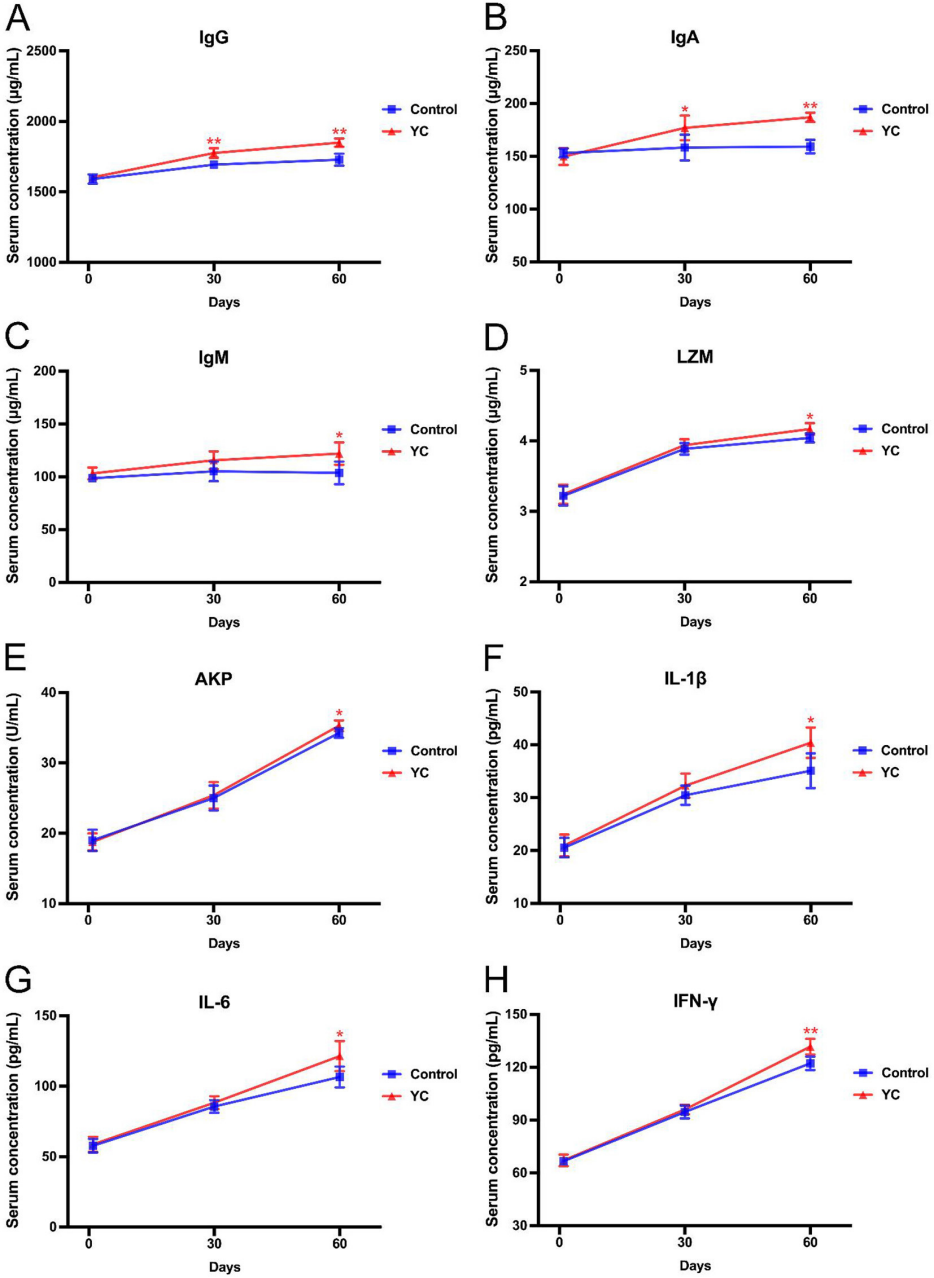
Effects of YC on the indexes related to immune function of beef cattle. **(A)** Effects on the serum IgG concentration. **(B)** Effects on the serum IgA concentration. **(C)** Effects on the serum IgM concentration. **(D)** Effects on the serum LZM concentration. **(E)** Effects on the serum AKP concentration. **(F)** Effects on the serum IL-1β concentration. **(G)** Effects on the serum IL-6 concentration. **(H)** Effects on the serum IFN-γ concentration. *p < 0.05, **p < 0.01. The value is the mean ± SD, *n* = 6.

As illustrated in Figure 2, there was no significant difference in serum levels of IgG, IgA, or IgM in beef cattle between the two groups at the beginning of the experiment. As the experiment progressed, the beef in YC group showed significantly higher serum levels of IgG and IgA than those in the control group on day 30 (*P* < 0.05), which increased by 7% and 17.3% respectively on day 60 (*P* < 0.01). Moreover, the IgM content in YC group increased by 17.65% on the 60th day (*P* < 0.05).

As to any one of the five other indexes of serum immune function of the beef cattle, there was no significant difference between the control group and YC group, either on the 1st day or on the 30th day (*P* > 0.05), but both showing an upward trend. On the 60th day, the LZM content and AKP activity, as well as the serum levels of IL-1β, IL-6, and IFN-γ in YC group were evidently elevated by 0.12 μg/ml, 1.03 U/ml, 5.3 pg/ml, 14.82 pg/ml, and 9.54 pg/ml, compared to the control group (*P* < 0.05), with increases of 2.97%, 3.01%, 15.09%, 13.90%, and 7.81%, respectively.

### 3.4 Effects of YC on the intestinal microbiota structure in beef cattle

**Figure 3:**
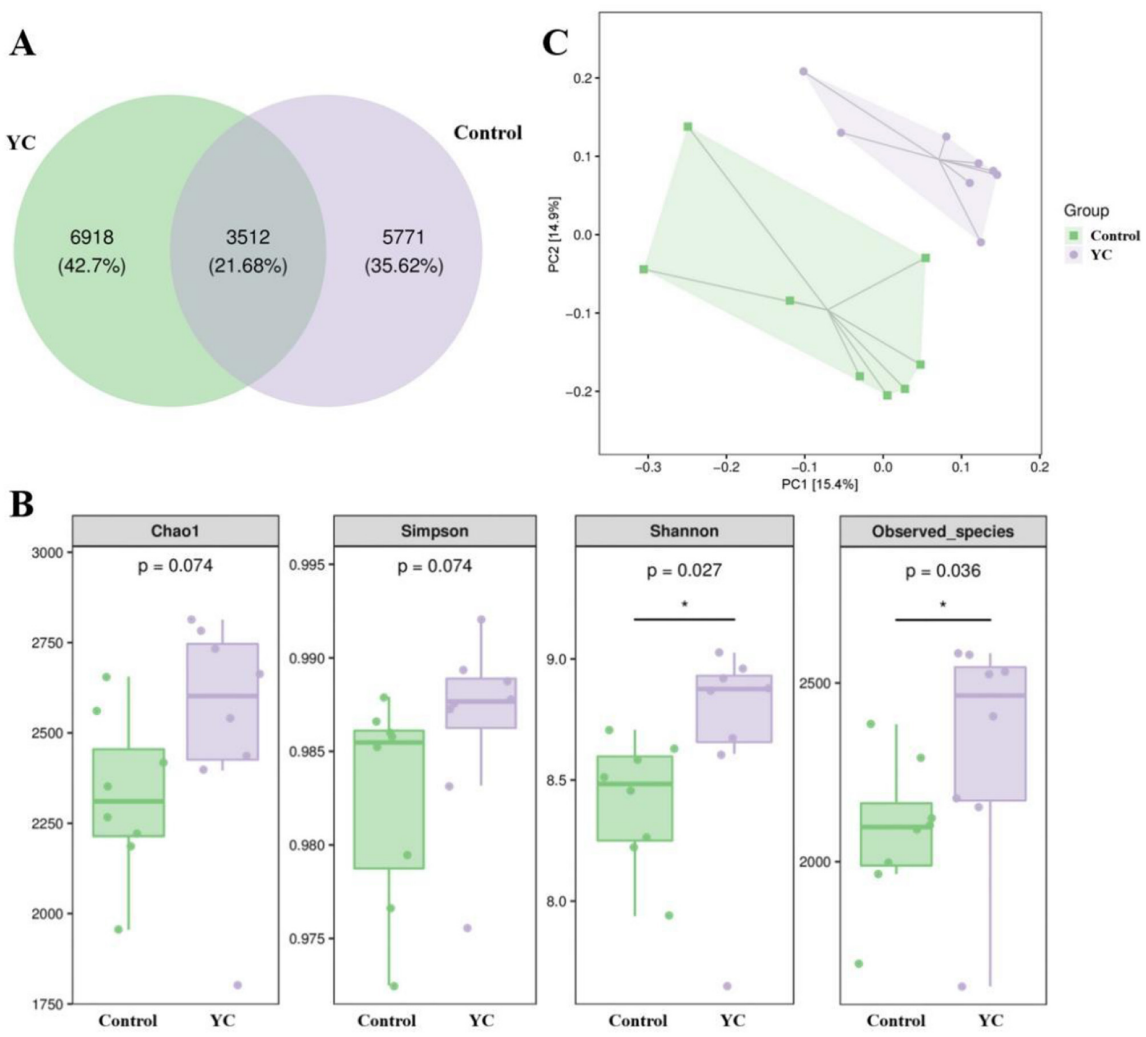
Diversity of intestinal microbiota in beef cattle. **(A)** Venn diagram highlighting the overlapping ASVs when comparing composition of gut microbiota two groups. **(B)** α diversity analysis. **(C)** β diversity analysis. *n* = 8. ^*^p < 0.05, ^**^p < 0.01.

In order to investigate the possible effects of yeast culture on the gut microbiota structure of beef cattle, 16s rRNA gene sequencing was performed using primers specific for the V3–V4 region. In total 614,688 effective sequences were obtained across 16 samples (average: 38,418 sequences/sample), leading to the identification of 16201 ASVs at the 97% nucleotide sequence identity level (Figure 3A). In total, 3,512 ASVs were represented across samples in different groups, while the control and YC groups, respectively, harbored 5,771 (35.62%) and 6,918 (42.7%) unique ASVs.

Alpha diversity indices revealed that the coverage rate of the tested samples reached over 99.5%, indicating that the size and depth of the sequencing samples was available to measure the true microbial community in fecal samples. From Figure 3B, we can see that Shannon index (the microbial diversity index) and Observed species index (the microbial richness index) of the samples in YC group were distinctly higher than those in the control group (*P* < 0.05).

Analyses of β-diversity and principal co-ordinates analysis (PCoA) were performed on the fecal microbiota structure of beef cattle using the Bray Curtis distance (Figure 3C). The sample points of the two groups were apparently separated, and intestinal microbiota structure in the same group showed a clear clustering trend, representing visible differences in species composition between them.

**Figure 4:**
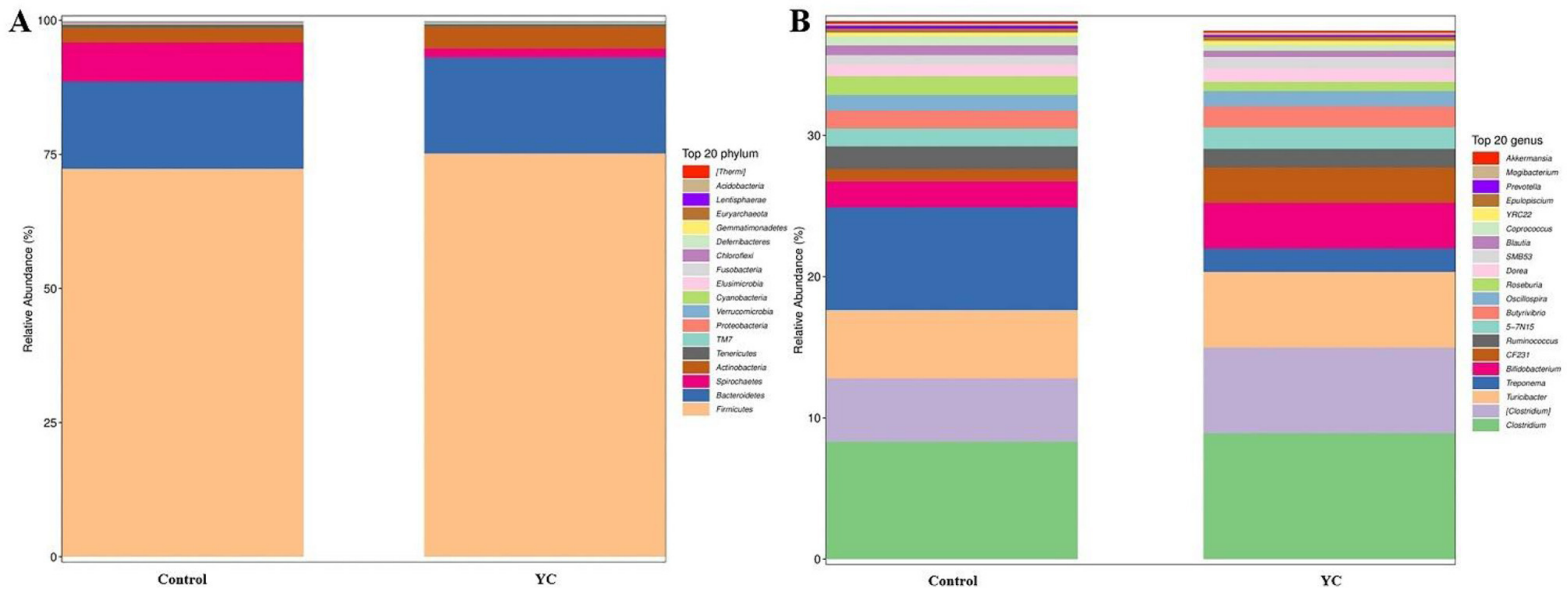
Composition of the intestinal microbiota in the beef cattle. The composition of the intestinal microbiota in the control and YC groups at the phylum **(A)** and genus **(B)** levels. Control = basal diet. YC = basal diet supplemented with yeast culture. *n* = 8.

**Figure 5:**
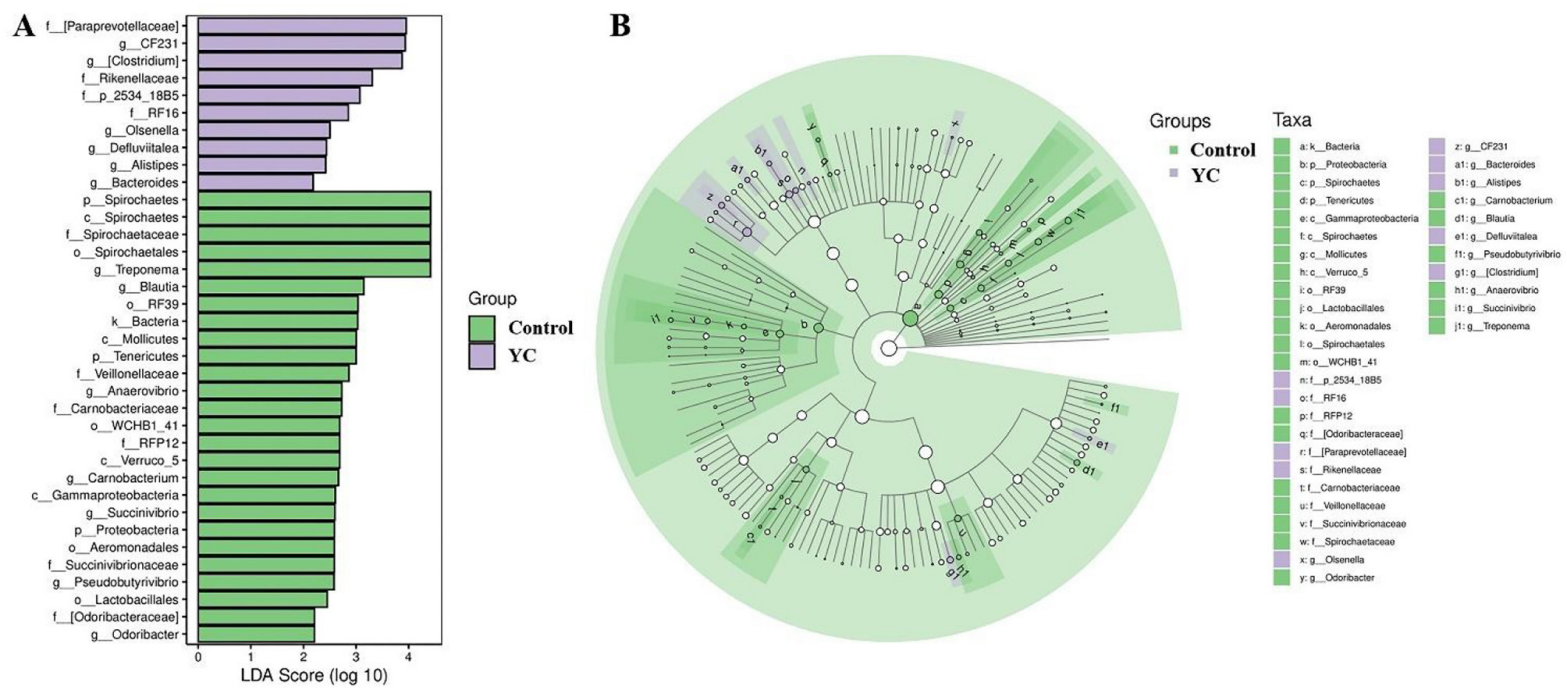
Linear Discriminant Analysis (LDA) on the genus level. Bacterial taxa at the genus level significantly identified by linear discriminant analysis coupled with effect size (LEfSe) using the default parameters between groups control and YC. **(A)** Bar Chart. **(B)** Circular Tree Diagram. Control = basal diet. YC = basal diet supplemented with yeast culture. *n* = 8.

*Firmicutes, Bacteroidetes*, and *Spirochaetes* were the predominant phyla in control and YC groups samples (Figure 4A), while the predominant genera were *Clostridiaceae_Clostridium, Peptostreptococcaceae_Clostridium*, and *Turicibacter* (Figure 4B). The LEfSe (LDA Effect Size) analysis results showed that when the LDA effect size was ≥3 and the *P*-value was < 0.05, the YC group exhibited a significant increase in the relative abundance of *Paraprevotellace_CF231* and *Peptostreptococcaceae_Clostridium* at the genus level, while the relative abundance of *Spirochaetaceae_Treponema* and *Lachnospiraceae_Blautia* decreased significantly (Figures 5A, B).

### 3.5 Analyses of correlations between intestinal microbiota, antioxidant capacity, and immune function indicators

**Figure 6:**
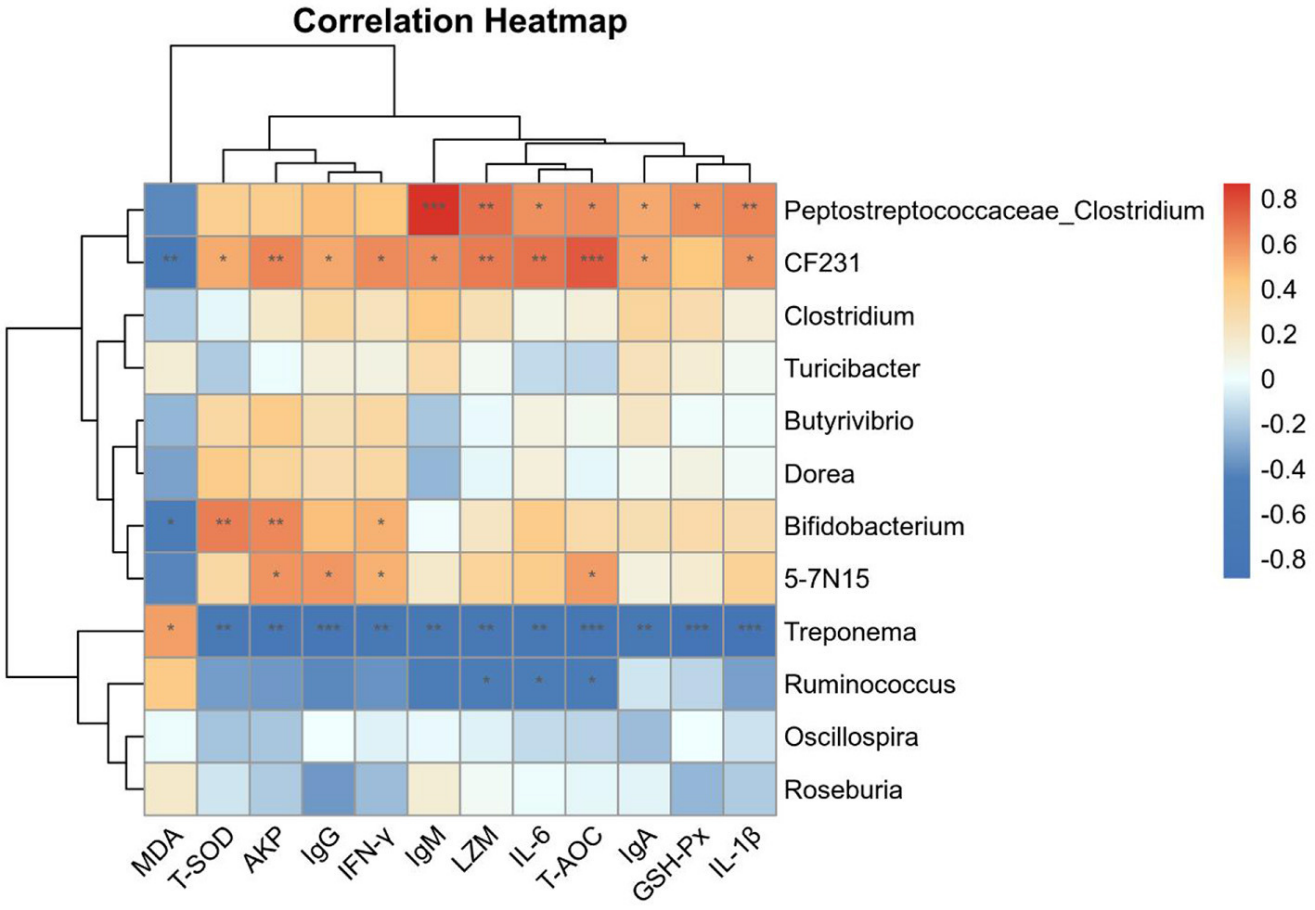
Thermogram showing Spearman correlations of the relationships between intestinal bacteria, the antioxidant capacity, and immune function indicator content. Positive and negative correlations are shown in red and blue, respectively. *p < 0.05, **p < 0.01, ***p < 0.001. *n* = 8.

Correlations between serum antioxidant capacity, immune function indicators, and the dominant genera detected in fecal samples were next assessed. Significant positive correlations were observed between *Peptostreptococcaceae*_*Clostridium* abundance and the serum levels of IgM, LZM, and IL-1β (*P* < 0.01). Noticeable positive correlations were observed between *Paraprevotellaceae_CF231* abundance and the levels of T-AOC, LZM, AKP, and IL-6 (*P* < 0.01), while there was an obvious negative correlation between the former and MDA levels (*P* < 0.01). Significant negative correlations were observed between *Spirochaetaceae_Treponema* abundance and the levels of T-SOD, T-AOC, GSH-Px, IgG, IgA, IgM, LZM, AKP, IL-1β, IL-6, and IFN-γ (*P* < 0.01; Figure 6). These data indicate that YC supplementation and associated modulation of the intestinal microbiota may contribute to the improvements of antioxidant capacity and immune function in beef cattle.

## 4 Discussion

Probiotics and their cultures are widely used in human and a variety of animal industries to improve growth performance, health condition, gastrointestinal microbial balance, and intestinal morphology (16, 17). At present, there are various types of yeast cultures and other probiotic microbial preparations, but different fermentation processes, substrates, and strains can affect the quality and application effectiveness of the cultures. YC used in this study, including the screening of yeast strains, selection of fermentation substrates, and optimization of fermentation processes, was independently developed by our research team, which showed outstanding advantages such as comprehensive nutritional content, diverse metabolites, and mature production processes (18, 19). In this paper, we evaluated the effects of YC as a feed supplement on a range of parameters associated with growth and health, including growth performance, immune function, antioxidant capacity and intestinal microbiota structure in Simmental beef cattle.

### 4.1 Effects of YC on the growth performance of beef cattle

It is well-known that the level of feed intake and the ability to digest and absorb nutrients directly affect the growth performance of animals. A large number of studies revealed that feeding YCs improved the growth performance, rumen development and feed efficiency in calves, particularly in pre-weaning calves (20). Geng et al. found that adding YC to TMR significantly promoted the growth performance of bulls, as evidenced by increased average weight by 10 kg and ADG by 0.1 kg in the YC-treated group, compared to the control group (21). In the same way, the results of this study showed that the YC addition in the cattle feed obviously improved ADF and ADG by 5.50% and 11.36%, respectively, while F/G was depressed by 5.33%, during the 30th to 60th days, indicating that YC could promote the growth performance of beef cattle.

In addition, the beef cattle in both groups were fed with an appropriate amount of concentrated feed such as corn. The feces in YC group were soft and shaped, with a pile height of 4–5 cm, a ring around, a cavity in the middle, and no obvious corn grains were seen, while those in the control group were loose, thin and shapeless, with a pile height of < 2.5 cm, scattered when falling to the ground, and undigested corn grains were visible in the feces. Moreover, our previous results *in vitro* showed that YC can increase the concentrations of total VFA, acetic acid, propionic acid, butyric acid, ammonia nitrogen and other nutrients, and it could enhance the digestibility of dry matter, crude protein, neutral detergent fiber and acid detergent fiber of the diet (10). These clinical manifestations and laboratory results further indicated that YC may promote the growth performance by improving the digestion and absorption of nutrients in the gastrointestinal tract, as well as the rumen fermentation function and digestibility of dietary nutrients.

### 4.2 Effects of YC on the antioxidant capacity of beef cattle

Due to the high concentrated diet, beef cattle in the growth period grow rapidly, and thus metabolic activity may remain elevated and produce a lot of free radicals. Therefore, beef cattle in such a period are prone to free radical damage, leading to many problems, such as decreased growth performance, elevated F/G ratio, and increased disease morbidity. The enzyme system scavenging free radicals, including T-SOD and GSH-Px, can maintain a dynamic balance of oxidation-reduction (22). T-SOD can protect cell membranes from damage by eliminating superoxide anion free radicals. GSH-Px can remove reactive oxygen intermediates and protect the macromolecular components of tissues from the invasion of oxygen free radicals. MDA is a biomarker of lipid peroxidation, whose content directly reflects the degree of lipid peroxidation and free radical metabolism in the body. In our previous reports, dietary YC supplementation significantly increased the serum levels of T-AOC, SOD, and GSH-Px in Mongolian lambs (10). In this study, the activities of T-SOD, GSH-PX, and T-AOC in the cattle serum in YC group were remarkably higher than those in the control group on the 60th day, while the MDA content in the former group was evidently lower than that in the latter group. These results indicated that YC can significantly reduce response to oxidative stress and decelerate the oxidative damage in beef cattle.

Interestingly, the cattle herd encountered a stress challenge caused by sudden temperature drops during the test period, resulting in decrease in feed intake and occurrence of diarrhea. However, the decrease in feed intake was not obvious in YC group, and the number of diarrhea cases was also lower than that in the control group, which further confirmed that YC can enhance the anti-stress ability of beef cattle and alleviate the stress caused by the external environment.

### 4.3 Effects of YC on the immune function of beef cattle

Immunoglobulin is the main antibody that mediates humoral immunity, and the elevation of antibodies in serum can to some extent reflect the enhancement of the body's immune function. IgG has the highest content in serum immunoglobulin and plays a major role in specific immunity; IgM is the first antibody produced in the humoral immune response induced by antigen stimulation, and plays an important role in early defense of the body; IgA is the main antibody in mucosal immunity (23). In this study, IgG, IgM, and IgA in the serum of beef cattle in YC group were significantly increased, indicating that YC enhanced the specific humoral immunity of beef cattle. Research has shown that lambs with YC added in the diet have higher serum concentrations of IgM and IgG (10), which is consistent with the findings of this study. One of the reasons may lie in that probiotics in YC can activate relevant lymphoid tissues in the intestinal mucosa, stimulate the secretion of immunoglobulins, improve immune recognition ability, induce immune cells to produce cytokines, activate the systemic immune system, and enhance the body's immune function (24).

LZM is a kind of hydrolase secreted by monocytes that can dissolve bacterial cell wall, and AKP is one of the marker enzymes of macrophage lysosomes. Both play positive roles in maintaining non-specific immune balance and resistance to diseases by enhancing the phagocytic and bactericidal abilities of phagocytes in animals (25, 26). In our study, the LZM activity and AKP content in the serum of beef cattle fed with YC increased by 2.97% and 3.01%, respectively, indicating that YC could improve the non-specific immune function and thus enhance the resistance of beef cattle. Cytokines are hormone-like protein molecules produced by the activated lymphocytes and monocytes. Although cytokines are relatively low in animal serum, they play important roles in the defense against diseases (27). IL-1β and IL-6 can promote the proliferation and differentiation of immune cells and enhance their functional activity (28, 29). IFN-γ is an important immune factor with functions of antiviral, enhancing phagocytosis of macrophages, and regulating immunity (30). Our results illustrated that the contents of IL-1β, IL-6, and IFN-γ in beef cattle serum were significantly increased by YC addition on the 60th day. Chen et al. also reported that feeding YC could obviously enhance the serum levels of IL-6, IL-1β, and IFN-γ in lambs (10), which supports the conclusion of this research.

Evidence has shown that the dietary addition of yeast products could affect immune response in animals, and the mechanism may be related to that yeast cell components can effectively stimulate immunocyte activation at a physiologically reasonable concentration (31, 32). For example, β-glucan and mannan could bind to surface mode receptors of cells like macrophages and neutrophils, which activated such phagocytes and regulated the expression of antioxidant factors and inflammatory factors (32).

### 4.4 Effects of YC on the intestinal microbiota structure of beef cattle

Analyzing fecal samples by high-throughput sequencing technology can more accurately reflect changes in the composition and diversity of gastrointestinal microbiota in humans and animals, which plays an important role in the health and growth of hosts. The Observed species indexes focus on reflecting the richness of biological communities, while Shannon indexes focus on reflecting the microbial diversity. In our study, the Shannon index and Observed species index of the beef fecal samples in YC group were significantly higher than those in the control group. These data indicated that YC addition increased the diversity and richness of the fecal microbiota.

Researchers have reported that the microbial composition of calf feces was basically similar at the phylum level, with *Firmicutes* and *Bacteroidetes* being the dominant phyla, accounting for over 80% of the total bacteria (33), consistent with the results of this study. *Firmicutes* mainly participate in the metabolism and absorption of carbohydrates and proteins (34), while *Bacteroidetes* are principally involved in the degradation of non-fibrous substances and polysaccharides, as well as facilitating the absorption of feed nutrients, and therefore improving the nutrient digestibility (35). In addition *Bacteroidetes* could promote the development of the gastrointestinal immune system (36).

We found that adding YC to the diet significantly increased the relative abundance of *Bacteroidetes_ParaPrevotellace_CF231* and *Firmicutes_Peptostreptococaceae_Clostridium* in the intestinal tract of beef cattle. Since both *CF231* and *Peptostreptococaceae_Clostridium* could ferment indigestible carbohydrates, short chain fatty acids (SCFAs) are produced to maintain the acidic environment of the intestine, inhibit the colonization of pathogens, and have anti-inflammatory and protective barrier functions (37, 38). In addition, studies have shown that both the two bacteria may participate in regulating the host's immune response through their metabolites and cellular components, and play a key role in the development and maturation of the intestinal mucosal immune system (39). The above information demonstrated that YC may enhance the degradation of non-cellulose and polysaccharides by promoting the growth of microorganisms such as *CF231* and *Peptostreptococcaceae_Clostridium*, thus promoting the digestion and absorption of nutrients by the gastrointestinal tract. As previously mentioned, YC could elevate the growth performance of the beef cattle, which may be due to such above mechanisms to some extent. Moreover, the abundance of *Clostridium* and *CF231* was positively associated with the levels of IgM, LZM, IL-1β, IL-6, AKP, and T-AOC, which further indicate that the YC efficacy on improving immune function in beef cattle may be related to the increase of the relative abundance of these bacteria above-mentioned. On the other hand, *Spirochaete* phyla comprise various pathogenic bacteria (40, 41), which cause adverse effects on the colonization of early gut microbiota in calves (42) and are closely associated with gut inflammation and potential induction of diarrhea (43), resulting in decreased growth performance and productivity. In our research, the relative abundance of *Spirochetes_Treponema* in fecal samples of cattle additionally fed with YC was obviously lower than that of the control group, indicating that YC may reduce the numbers of pathogenic bacteria and promote the colonization of beneficial microorganisms in the intestine by repressing the contents of *Treponema*. Moreover, the abundance of *Treponema* was negative correlated with the levels of T-SOD, T-AOC, GSH-Px, IgG, IgA, IgM, LZM, AKP, IL-1β, IL-6, and IFN-γ. The above results further explain the enhancing effects of YC on the growth performance, immune function and antioxidant capacity of beef cattle may be partly due to its regulation on the gut microbiota structure.

This present paper studied the effects of YC on the production performance, immune function, antioxidant capacity, and gut microbiota structure of Simmental beef cattle at a macro level. We previously demonstrated that both YC and one of its main active ingredients, β-glucan, added to the sheep diet could improve the growth performance, immune function and antioxidant capacity of Mongolian sheep, indicating that YC effects may be due to its rich variety of nutrients, especially such as polysaccharides β-glucan and mannan (10). However, there is currently a lack of deep research on the corresponding mechanisms, which is also the focus of our team's ongoing research. Perhaps some new research methods and data can provide assistance, such as using spatial co-indexing of transcriptomes and epitopes (CITE) sequencing, spatial ATAC–RNA–Protein (DBiT ARP) sequencing, or Perturb-DBiT to explore the mechanisms of YC driving the growth and development of the gastrointestinal tract and their microbiota structure, as well as the development and activation of immune tissues in both physiological and pathological levels (44–46).

## 5 Conclusion

The effects of YC on growth performance, antioxidant capacity, immune function, and intestinal microbiota structure in Simmental beef cattle were evaluated *in vivo* in the present study. Results showed that YC added in diet could improve the growth performance, antioxidant capacity, and immune function of beef cattle, and improve the intestinal microbiota structure as well. Therefore, YC could be used as an animal feed additive and be considered as a potential substitute for dietary subtherapeutic antibiotics. These data provided a theoretical support for the clinical application of this yeast fermented preparation in cattle breeding husbandry.

## Data Availability

The datasets presented in this study can be found in online repositories. The names of the repository/repositories and accession number(s) can be found in the article/supplementary material.
